# Crop rotation and tillage management options for sustainable intensification of rice-fallow agro-ecosystem in eastern India

**DOI:** 10.1038/s41598-020-67973-9

**Published:** 2020-07-07

**Authors:** Rakesh Kumar, Janki Sharan Mishra, Karnena Koteswara Rao, Surajit Mondal, Kali Krishna Hazra, Jaipal Singh Choudhary, Hansraj Hans, Bhagwati Prasad Bhatt

**Affiliations:** 1 ICAR-Research Complex for Eastern Region, Patna, Bihar 800 014 India; 2 ICAR-Indian Institute of Pulses Research, Kanpur, Uttar Pradesh 208 024 India; 3 ICAR-RCER, Research Centre, Plandu, Ranchi, Jharkhand 834 010 India

**Keywords:** Climate sciences, Environmental sciences

## Abstract

Presently, rice-fallows are targeted for cropping intensification in South Asia. Rice**-**fallows a rainfed mono-cropping system remain fallow after rice due to lack of irrigation facilities and poor socio-economic condition of the farmers. Nevertheless, there is the scope of including ecologically adaptable winter crops in water-limited rice-fallow conditions with effective moisture conservation practices. The study aimed to identify the winter-crops that are adaptable and productive in rice-fallow conditions and to evaluate the different tillage-based crop establishment practices for soil moisture conservation, grain yield, economics, and sustainability parameters. Six different crop establishment and residue management (CERM) practices viz*.,* zero**-**tillage direct seeded rice (ZTDSR), zero-tillage transplanted rice (ZTTPR), puddled transplanted rice (PTR), ZTDSR with rice residue retention (ZTDSR_R+_), ZTTPR with rice residue retention (ZTTPR_R+_), PTR with rice residue retention (PTR_R+_) as main-plot treatment and five winter crops (chickpea, lentil, safflower, linseed, and mustard) as sub-plot treatment were evaluated in a split-plot design. The productivity of grain legumes (chickpea and lentil) was higher over oilseed crops in rice-fallow conditions with an order of chickpea > lentil > safflower > mustard > linseed. Among the CERM practices, ZTDSR_R+_ and ZTDSR treatments increased the grain yield of all the winter crops over PTR treatment, which was primarily attributed to higher soil moisture retention for an extended period. Grain yield increment with conservation tillage practices was highly prominent in safflower (190%) followed by lentil (93%) and chickpea (70%). Rice grain yield was higher (7–35%) under PTR treatment followed by ZTDSR treatment. Conservation tillage practices (ZTDSR, ZTTPR) reduced energy use (11–20%) and increased the energy ratio over conventional tillage practice (PTR), higher in rice-safflower, rice**-**lentil and rice**-**chickpea rotations. Higher net return was attained in rice**-**safflower and rice**-**chickpea rotations with ZTDSR_R+_ treatment. Predicted emission of greenhouse gases was markedly reduced in ZTDSR treatment (30%) compared to ZTTPR and PTR treatments. Hence, the study suggests that cropping intensification of rice-fallows with the inclusion of winter crops like chickpea, lentil, and safflower following conservation tillage practices (ZTDSR_R+_ in particular) could be the strategic options for achieving the higher system productivity, economic returns, and energy use efficiency with the reduced emission of greenhouse gases.

## Introduction

Rice**-**fallows, the rainfed mono**-**crop production system, nowadays are gaining greater attention for cropping intensification in South Asia^1^. Rice**-**fallow areas extended over 22.3 M ha in South Asia, having maximum acreage in India (88.3%) and remaining in Bangladesh (8.7%), Pakistan (0.5%), Sri Lanka (1.1%), Nepal (1.4%), and Bhutan (0.02%)^2^. The major challenges of cropping intensification in rice-fallow areas includes lack of irrigation facilities, predominance of the long**-**duration rice cultivars that delays winter crop establishment, higher evaporative loss of the residual soil moisture, and poor financial status of the farming community^3, 4^. Plant stress emanating from the soil moisture deficit severely affects the crop establishment and productivity of winter crops in rice-fallow areas and often leads to the total crop failure^5, 6^.

Rice-fallows offer an extensive scope to increase the country’s winter crop area through strategic crop management practices^7, 8^. The choice of an appropriate winter crop is crucial and crop must have the stress-adaptive traits to withstand the biotic and abiotic stresses particular to the rice**-**fallow conditions^9–11^. Early maturity, fast growth, early ground cover and deep**-**roots have been suggested as desirable plant characters for water-limited rice**-**fallow conditions^12, 13^. Grain legumes (lathyrus, chickpea, lentil) and oilseed crops (safflower, mustard and linseed) can be grown in rice**-**fallows. However, the productivity and profitability of these winter crops are still uncertain in rice-fallow areas of eastern India.

Sustainability of rice-fallows in eastern India is further challenged by the soil quality degradation, declining groundwater resources, environmental pollution and diminishing farm profitability. In South Asia, wet**-**tillage (puddling) is widely practiced for rice crop establishment that adversely affects the productivity of subsequent winter crop in rice**-**fallows^14, 15^. The negative impact of tillage-intensive conventional puddled transplanted rice (PTR) on soil quality is well documented^9, 12, 16^. Agro**-**techniques that conserve the residual soil moisture and maintain a favourable soil environment may alleviate moisture-deficit stress in rice**-**fallows^16^. Conservation tillage practices such as zero**-**tillage transplanting and zero**-**tillage direct seeding in rice are likely to improve soil hydrology and crop water use efficiency^12^. Crop residue retention has multiple advantages including soil moisture conservation, weed control, and sustaining soil microbial activity^14^. Now, advanced agro-tools (e.g. Happy Seeder) are also developed for the sowing of crops under the heavy load of crop residues^15^.

Under these backgrounds, a 2**-**year field experiment was conducted for evaluating the performance of different winter crops in rice**-**fallow conditions of the eastern India under different tillage-based crop establishment cum residues management (CERM) practices. The major hypotheses of the present study were (1) conservation tillage practices with crop residue retention can conserve the residual soil moisture for a longer period and increase winter crop productivity in rice-fallows, (2) in rice-fallows, grain legumes (chickpea, lentil) with improved plant traits are likely to be more adaptable and productive over linseed and mustard, (3) energy productivity, production economics and greenhouse gases (GHGs) emission are the indicators of sustainability and vary widely with crop rotation and management practices in rice-fallows. Thus, the findings of the study could be useful for sustainable intensification of rice-fallow areas with strategic interventions.

## Methods

### Study site

The field experiment was conducted during 2016**–**2018 in the rice-fallow experimental plot of ICAR-Research Complex for Eastern Region, Patna (25° 35*′* N, 85° 05*′* E and 51 m above sea level). The climate of the site is sub-tropical humid. Rainfall, evaporation, minimum and maximum temperatures during the experimental period were recorded and are presented in Supplementary Fig. 1. During both the year, winter season (November–April) received less than 30 mm of rainfall and considered as dry, whereas, monthly mean pan evaporation (E_pan_) ranged between 32–263 mm in 2016**–**2017 and 23–120 mm in 2017**–**2018. The soil samples were collected through core method of soil sampling and initial physico-chemical properties were determined by the standard methods described by various workers (Supplementary Table 1). The soil was silty**-**clay loam in texture, pH 7.58, bulk density 1.63 g cm^–3^, and organic carbon 5.6 g kg^–1^. Available nutrients status of soil is presented in Supplementary Table 1.

### Experimental design

Six different crop establishment and residue management (CERM) treatments (zero**-**tillage direct seeded rice (ZTDSR), zero**-**tillage transplanted rice (ZTTPR), puddled transplanted rice (PTR), ZTDSR with rice residue retention (ZTDSR_R+_), ZTTPR with rice residue retention (ZTTPR_R+_), PTR with rice residue retention (PTR_R+_)] were randomized in main-plots and five winter crops [chickpea (*Cicer arietinum* L.), lentil (*Lens culinaris* L.), safflower (*Carthamus tinctorius* L.), linseed (*Linum usitatissimum* L.), and mustard (*Brassica juncea* L.)] were randomly allotted in sub-plots. The experimental layout was a split-plot design with three replications. The detail of CERM treatments is given in Table 1. In the residue retention treatments (ZTDSR_R+_, ZTTPR_R+_ and PTR_R+_), crops were harvested manually leaving ~ 20 cm rice stubble, whereas, in the residue removal treatments (ZTDSR, ZTTPR and PTR), rice crop was harvested manually close to the ground. The dimension of gross and net plot area was 13.0 m × 4.5 m and 12.5 m × 4.0 m, respectively.

**Table 1:** Treatment description and abbreviations used in the study.

Treatment abbreviation	Description
ZTDSR	Rice was directly sown (22.5-cm row spacing) in the main field under zero-tillage condition by Happy Seeder (zero–tillage seed cum fertilizer drill). A uniform seed rate of 30 kg ha^−1^ used in ZTDSR treatment (~ 90 plants m^−2^). Pre-established weeds were controlled through pre-sowing application of glyphosate (41% EC)
ZTDSR_R+_	Sowing and weed management were same as ZTDSR. Rice was manually harvested and 20-cm rice stubbles were retained as a part of the treatment
ZTTPR	Twenty-one day old seedlings were manually transplanted in unploughed main field with a spacing of 20 cm × 15 cm. One day before transplanting field was irrigated (~ 10 ha-cm) to make the soil soften and loose. Pre-established weeds were controlled through pre-sowing application of glyphosate (41% EC)
ZTTPR _R+_	Same as ZTTPR with 20-cm rice residues retention (~ 30%)
PTR	Field was prepared by two passes of dry-harrowing followed by planking. Wet-tillage was done by tractor-drawn rotavator under standing water (~ 10 cm). Manual transplanting of twenty-one day old rice seedling was done with a spacing of 20 cm × 15 cm
PTR _R+_	Same as PTR with 20-cm rice residue retention

### Crop management

Rice cultivar ‘Swarna Shreya’ (120 days duration) was used for the study. Rice seeds were treated with Tebuconazole (Raxil 60 FS) at 1 ml kg^−1^ seed for controlling of incidence of diseases. In ZTDSR and ZTDSR_R+_ treatments, pre**-**soaked (6 h) rice seeds were sown by zero**-**tillage seed drill at a row**-**spacing of 22.5 cm with a specific density of ~ 90 plants m^−2^. Rice nursery was raised on the same day of ZTDSR sowing. In PTR/PTR_R+_ treatments, the field was harrowed twice by disc harrow followed by planking. Then, wet-tillage (puddling) was done by tractor drawn puddler. Whereas under ZTTPR/ZTTPR_R+_ treatments, one day before transplanting, field plots were flooded with irrigation (~ 10 cm) and twenty**-**one day old seedlings were transplanted at a spacing of 20 cm × 15 cm. Three seedlings hill^−1^ were planted in PTR/ PTR_R+_ and ZTTPR/ZTTPR_R+_ treatments. Fertilizer N:P_2_O_5_:K_2_O at 120:60:40 kg ha^–1^ was applied to the rice crop. 50% N and 100% P and K as basal fertilizer were applied. The remaining 50% N was applied in two equal splits at tillering and panicle initiation stages.

After rice harvest, glyphosate (Roundup, 41% EC) at 1.5 l ha^−1^ was applied for controlling the weeds. Before sowing, seeds of all the winter crops were water-soaked overnight (12 h) for uniform germination. All the winter crops were sown under zero tillage condition by zero-till happy seeder. The cultivar and common agronomic practices of each winter crop are given in Table 2. As a part of the nutrient management, foliar spray of 2% urea was done in all the winter crops at flowering and grain development stages. One hand weeding was done at 35 days after sowing (DAS) to control the weeds. All winter crops were raised on residual soil moisture and no irrigation was applied to the winter crops.

**Table 2:** Common agronomic practices and cultivar used in the study.

Crop	Establishment method	Cultivar	Seed rate (kg ha^−1^)	Spacing (cm)	Sowing/transplanting time	Nutrient rate (N: P_2_O_5_: K_2_O kg ha^−1^)
Rice	ZTDSR	Swarna Shreya	30	22.5 × 5	June (3^a^)	120–60–40
	ZTTPR	Swarna Shreya	20	20 × 15	July (1)	120–60–40
	PTR	Swarna Shreya	20	20 × 15	July (1)	120–60–40
Chickpea		Pusa 256	80	22.5 × 5	October (4)	20–50–0
Lentil		HUL 57	40	22.5 × 5	October (4)	20–50–0
Safflower		PBNS 12	15	45 × 10	October (4)	30–20–20
Linseed		T 97	25	22.5 × 5	October (4)	50–30–20
Mustard		Proagro 5111	5	45 × 15	October (4)	40–20–20

^a^Value in parentheses represents the weeks of the corresponding month.

### Grain yield and yield attributes

A net plot area of 50 m^2^ was harvested separately for estimation of grain and straw/ stover yields. Grain yields were recorded after threshing and adjusted at ~ 12% moisture content (w/w). Rice equivalent yield (REY) of the winter crops was computed by converting their grain yield to rice yield with a price factor as per following formula^16^.

1 $${\text{REY}}({\text{kg}}\,{\text{ha}}^{ - 1} ) = \frac{{{\text{Grain}}\,\,{\text{yield}}\,\,{\text{of}}\,\,{\text{the}}\,\,{\text{winter}}\,\,{\text{crop}}({\text{kg}}\,\,{\text{ha}}^{ - 1} ) \times {\text{Price}}\,\,{\text{of}}\,\,{\text{winter}}\,\,{\text{crop}}\,\,({\text{INR}}\,\,{\text{kg}}^{ - 1} )}}{{{\text{Price}}\,\,{\text{of}}\,\,{\text{rice}}\,\,({\text{INR}}\,\,{\text{kg}}^{ - 1} )}}$$

The minimum support price of crops for the respective year [Indian rupee (INR)] was used for REY calculation.

The system rice equivalent yield (SREY) or annual system productivity and system production efficiency (SPE) were calculated by the following formula:

2 $$\begin{gathered} {\text{SREY}}\,\,({\text{kg}}\,\,{\text{ha}}^{ - 1} ) = {\text{Grain}}\,\,{\text{yield}}\,\,{\text{of}}\,\,{\text{rice}}\,\,({\text{kg}}\,\,{\text{ha}}^{ - 1} ) \hfill \\ + \frac{{{\text{Grain}}\,\,{\text{yield}}\,\,{\text{of}}\,\,{\text{the}}\,\,{\text{winter}}\,\,{\text{crop}}\,\,({\text{kg}}\,\,{\text{ha}}^{ - 1} ) \times {\text{Price}}\,\,{\text{of}}\,\,{\text{winter}}\,\,{\text{crop}}\,\,({\text{INR}}\,\,{\text{kg}}^{ - 1} )}}{{{\text{Price}}\,\,{\text{of}}\,\,{\text{rice}}\,\,({\text{INR}}\,\,{\text{kg}}^{ - 1} )}} \hfill \\ \end{gathered}$$

3 $${\text{System}}\,\,{\text{production}}\,\,{\text{efficiency}}\,\,({\text{kg}}\,\,{\text{ha}}^{ - 1} \,\,{\text{day}}^{ - 1} ) = \frac{{{\text{System}}\,\,{\text{rice}}\,\,{\text{equivalent}}\,\,{\text{yield}}\,\,({\text{kg}}\,\,{\text{ha}}^{ - 1} )}}{{{\text{Total}}\,\,{\text{duration}}\,\,{\text{of}}\,\,{\text{cropping}}\,\,{\text{system}}\,\,{\text{(days)}}}}$$

At maturity, ten rice plants (hill) were randomly selected for recording the observations on panicle length, number of grains panicle^−1^, effective tillers (panicle bearing tillers). Likewise, at maturity ten randomly selected plants of each winter crop were collected for taking measurements on plant height, number of pods plant^−1^ (chickpea and lentil), capsule plant^−1^ (linseed and safflower), and siliqua plant^−1^ (mustard). Random samples from harvested grains of each crop were taken in triplicate from each treatment and weight of 1,000 filled grains was weighed by analytical electronic balance and mean weight expressed in gram (g).

### Measurement of crop residue input

To estimate the quantity of recycled rice straw in residue retention plots, 1.0 m^2^ area were taken in each plot during crop harvest and the entire amount of residues was collected. Then, the collected residue was dried to a constant weight and expressed as t ha^−1^. Amount of rice straw retained in ZTDSR_R+_, ZTTPR_R+_, and PTR_R+_ treatments were ~ 7.2, 6.4, and 8.2 t ha^−1^, respectively.

### Soil moisture measurement

After the rice harvest, soil samples were collected from 0–15 and 15–30 cm depths at fifteen days interval using a core sampler and soil moisture content (w/w) were measured following the gravimetric method. Then, volumetric moisture content (v/v) was calculated by multiplying the soil moisture content (w/w) with bulk density^17^. The soil moisture depletion (cm) in different soil depths were calculated using the formula:

4 $${\text{Total}}\,\,{\text{soil}}\,\,{\text{moisture}}\,\,{\text{depletion}}\,\,{\text{(cm)}} = (\Sigma \Delta {\text{moisture}}\,\,({\text{g}}\,\,{\text{g}}^{ - 1} ) \times {\text{BD}}_{{\text{i}}} \times {\text{D}}_{{\text{i}}} ) + {\text{rainfall}}\,\,{\text{(cm)}}$$

where Δ = change in gravimetric soil moisture (g g^−1^) in two samplings intervals, BD_i_ = bulk density of ith layer of soil (g cm^-3^), d_i_ = depth of i^th^ layer of soil (cm).

The system water use efficiency was computed by dividing system productivity with the amount of water used in crop production (total rainfall + irrigation water applied to rice crop)^16^.

### Measurement of soil cracks

An area of 1.0 m × 1.0 m in each plot was selected for the measurements of crack width and depth. For the measurement of crack depth, a steel rod (1 mm diameter) was inserted at different points along the cracks and then averaged; slide caliper was used for measuring crack width^18^. Crack width measurement was done 1.0 cm below the soil surface to evade the amplified width caused by surface disturbance^19^. The crack volume in an area of 0.5 m × 0.5 m was measured by pouring down sand until all the cracks were filled up. The required sand volume to fill up the cracks was measured as crack volume. Six measurements were performed in each plot to even out the spatial variability.

### Economic analysis

All the fixed and variable costs (tillage, seed, fertilizers, pesticides, irrigation, harvesting) were taken into account for economic analysis. Labour cost used for land preparation, planting, irrigations, fertilization, pesticide application, and harvesting were based on the person-days ha^−1^ (8 h is equal to 1 person**-**day). Gross return was computed by multiplying grain and straw yields of component crops with their respective minimum support price (MSP) for the year, Government of India (Suppl. Table 2). The net return was computed as the difference between gross return and total cost of cultivation. The system net return was calculated by adding the net returns from both rainy and winter season crops in annual crop rotation.

### Energy budgeting

For estimation of energy inputs and outputs, a complete record of all the inputs (seed, fertilizer, agrochemicals, fuels, mechanical and machinery power) and outputs (grain and straw) were maintained. Inputs were translated from physical unit to the energy unit by multiplying with respective conversion coefficients (Suppl. Table 3). The energy use indices were calculated as suggested by Devasenapathy et al*.*^20^.

### Calculation of greenhouse gases (GHGs) emission and global warming potential (GWP)

Environmental impact of the different crop establishment methods and winter crops introduced under rice-fallow systems was assessed by calculating the emission of GHGs and GWP. GHGs emissions (N_2_O and CH_4_) were estimated indirectly in terms of CO_2_ equivalents. N_2_O and CH_4_ were converted into CO_2_ equivalent by multiplying with GWP equivalent factor 265 and 28 for N_2_O and CH_4_, respectively, for a timing edge of 100 years^21^. Seasonal emission of CH_4_ from puddle transplanted rice (PTR) and dry**-**seeded rice (DSR) was 12.8 and 5.6 kg season^−1^ ha^−1^, respectively as suggested by Padre et al*.*^22^. Emission of GHGs from nitrogen fertilizers was calculated with following the formula as suggested by Padre et al*.*^22^.

5 $${\text{N}}_{2} {\text{O}}\,\,{\text{emission}}({\text{kg}}\,\,{\text{ha}}^{ - 1} \,\,{\text{year}}^{ - 1} ) = \frac{{({\text{Factor}} \times 44 \times {\text{total}}\,\,N\,\,{\text{applied}}\,\,{\text{to}}\,\,{\text{the}}\,\,{\text{crop}})}}{(100 \times 28)}$$

where the emission factor for N_2_O is 0.51;

The greenhouse gas intensity (GHGI) was calculated by dividing system GWP with SREY (system productivity) and expressed as kg CO_2_ eq kg^–1^ SREY^23^.

### Statistical analysis

Data were subjected to analysis of variance (ANOVA) techniques to work out the significant differences among the treatments. Duncan’s multiple range test (DMRT) was used for comparison of treatment means at *p* = 0.05 in SAS 9.4 (Indian NARS Statistical Computing Portal). The data of parameters viz*.* system annual productivity (SREY), production economics, and energy budgeting were analyzed by split**-**plot ANOVA technique at* p* = 0.05. Principal component analysis (PCA) was performed using the window**-**based software PAST 3.14.

## Results

### Grain yield and yield attributes

**Figure 1:**
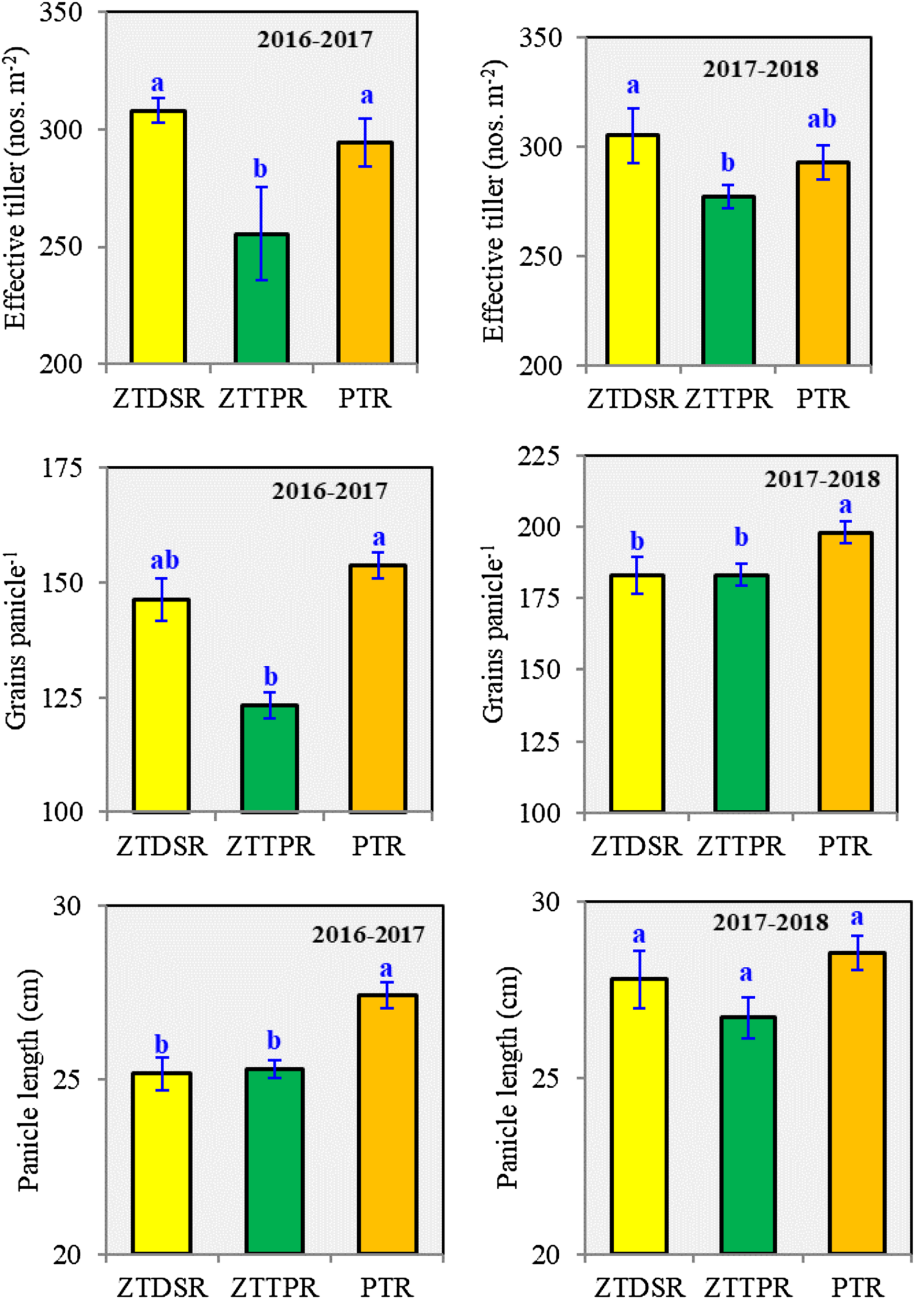
Effective tiller, panicle length, and grains panicle^−1^ of rice as influenced by different tillage based crop establishment practices. Error bar indicates standard error of mean. Different lowercase letters correspond to treatments are significantly different at *p* < 0.05 by Duncan’s multiple range test.

Among the CERM practices, ZTDSR_R+_ increased the grain yield (*p* < 0.05) of all the winter crops as compared to ZTTPR/ZTTPR_R+_ and PTR/PTR_R+_ treatments (Tables 3, 4). The order of treatments for average winter crop productivity was ZTDSR_R+_  > ZTDSR > ZTTPR_R+_  > ZTTPR > PTR_R+_  > PTR. Among the winter crops, grain yield of safflower was highly influenced by the CERM practices followed by lentil, chickpea, and linseed; whereas the effect was marginal on mustard. Crop residue retention increased the winter crops productivity by 14, 12 and 19% under ZTDSR, ZTTPR and PTR, respectively. Effect of CERM practices was prominent on plant growth and yield attributes i.e. plant height, pod/capsule/siliqua plant^−1^; and 1,000**-**grain weight, being higher under ZTDSR_R+_ treatment (Table 5). Rice grain yield was the highest in PTR treatment (Table 3). However, a reduction of 16–25% in rice yield was observed in ZTTPR compared to PTR and ZTDSR. The result showed that ZTDSR and PTR had higher (9%; *p* < 0.05) effective tillers over ZTTPR (Fig. 1). Higher grains panicle^−1^ (8%) and panicle length (8–9%) of rice were observed in PTR over ZTDSR and ZTTPR treatments.

**Table 3:** Grain and straw yields of rice as influenced by crop establishment and residue management (CERM) practices (2-year mean).

CERM practice	Grain yield (t ha^−1^)	Straw yield (t ha^−1^)
ZTDSR	4.60 ± 0.33^b^	5.74 ± 0.15^cd^
ZTDSR_R+_	4.90 ± 0.28^ab^	5.98 ± 0.35^c^
ZTTPR	3.89 ± 0.14^c^	5.56 ± 0.26^cd^
ZTTPR _R+_	4.05 ± 0.16^c^	5.36 ± 0.22^d^
PTR	5.30 ± 0.15^a^	7.38 ± 0.17^a^
PTR _R+_	5.22 ± 0.18^a^	6.48 ± 0.29^b^

Different letters in a column are significantly different at *p* < 0.05 according to Duncan’s multiple range test.

**Table 4:** Grain yields of winter crops as influenced by different tillage cum crop establishment methods and residues management (CERM) practices (2-year mean).

CERM practice	Grain yield (t ha^−1^)				
	Chickpea	Lentil	Safflower	Linseed	Mustard
ZTDSR	1.66 ± 0.11^ab^	1.61 ± 0.11^a^	1.66 ± 0.07^b^	0.80 ± 0.05^b^	1.11 ± 0.05^a^
ZTDSR_R+_	1.84 ± 0.13^a^	1.76 ± 0.12^a^	1.89 ± 0.08^a^	1.04 ± 0.07^a^	1.13 ± 0.07^a^
ZTTPR	1.28 ± 0.08^cd^	1.14 ± 0.07^bc^	0.95 ± 0.04^d^	0.80 ± 0.05^b^	0.95 ± 0.07^a^
ZTTPR _R+_	1.46 ± 0.08^bc^	1.17 ± 0.07^b^	1.17 ± 0.06^c^	0.86 ± 0.06^b^	1.05 ± 0.08^a^
PTR	1.08 ± 0.06^d^	0.91 ± 0.06^c^	0.65 ± 0.04^e^	0.62 ± 0.04^c^	0.92 ± 0.06^a^
PTR _R+_	1.21 ± 0.08^cd^	1.05 ± 0.07^bc^	0.75 ± 0.03^e^	0.79 ± 0.04^b^	1.14 ± 0.09^a^

Different letters in a column are significantly different at *p* < 0.05 according to Duncan’s multiple range test.

**Table 5:** Growth and yield attributes of winter crops as influenced by crop establishment methods and residues management (CERM) practices (2-year mean).

Parameter	CERM practice	Chickpea	Lentil	Safflower	Linseed	Mustard
Pod/capsule/siliqua (nos. plant^−1^)	ZTDSR	66.3 ± 2.98^a^	260.7 ± 11.72^a^	17.2 ± 0.60^a^	46.6 ± 1.02^ab^	274.8 ± 12.36^ab^
	ZTDSR_R+_	67.9 ± 3.61^a^	264.1 ± 14.03^a^	17.9 ± 0.45^a^	50.2 ± 1.55^a^	297.0 ± 15.78^a^
	ZTTPR	62.9 ± 3.85^a^	207.6 ± 12.72^b^	13.9 ± .59^b^	50.5 ± 1.66^a^	256.0 ± 15.69^bc^
	ZTTPR _R+_	65.9 ± 3.00^a^	222.7 ± 10.12^b^	15.1 ± 0.39^b^	49.5 ± 1.50^a^	244.2 ± 11.10^bc^
	PTR	63.4 ± 2.69^a^	162.6 ± 6.90^c^	7.5 ± 0.21^d^	43.0 ± 1.41^b^	241.2 ± 10.23^bc^
	PTR _R+_	63.8 ± 0.80^a^	170.3 ± 2.12^c^	10.1 ± 0.26^c^	49.7 ± 1.07^a^	228.2 ± 2.85^c^
Plant height (cm)	ZTDSR	38.1 ± 2.00^bc^	32.3 ± 1.45^b^	116.7 ± 5.37^a^	61.0 ± 3.48^a^	161.5 ± 7.26^a^
	ZTDSR_R+_	42.1 ± 1.79^ab^	40.7 ± 2.16^a^	129.0 ± 6.05^a^	63.8 ± 2.93^a^	164.7 ± 8.75^a^
	ZTTPR	36.2 ± 1.40^c^	30.1 ± 1.84^bc^	82.6 ± 3.07^c^	59.6 ± 1.89^a^	147.9 ± 9.06^a^
	ZTTPR _R+_	41.4 ± 1.35^ab^	31.6 ± 1.44^bc^	95.5 ± 3.81^b^	59.9 ± 1.68^a^	166.3 ± 7.56^a^
	PTR	34.5 ± 0.99^c^	28.1c ± 1.19^c^	69.8 ± 1.94^c^	47.6 ± 1.31^b^	133.1 ± 5.65^a^
	PTR _R+_	42.6 ± 1.19^a^	30.1 ± 0.38^bc^	73.9 ± 1.86^c^	49.9 ± 1.39^b^	140.9 ± 1.76^a^
1,000-grain weight (g)	ZTDSR	275.8 ± 12.40^a^	19.9 ± 0.89^a^	53.7 ± 1.60^a^	7.47 ± 0.17^a^	7.70 ± 0.35^b^
	ZTDSR_R+_	291.5 ± 15.49^a^	20.6 ± 1.09^a^	55.9 ± 1.21^a^	8.29 ± 0.19^a^	8.70 ± 0.46^a^
	ZTTPR	271.4 ± 16.63^a^	18.6 ± 1.14^a^	48.0 ± 1.12^b^	6.96 ± 0.24^a^	5.60 ± 0.34^c^
	ZTTPR _R+_	282.0 ± 12.82^a^	19.7 ± 0.90^a^	52.2 ± 0.97^a^	7.59 ± 0.32^a^	5.80 ± 0.26^c^
	PTR	253.2 ± 10.74^a^	18.3 ± 0.78^a^	43.9 ± 1.23^b^	7.27 ± 0.18^a^	5.50 ± 0.23^c^
	PTR _R+_	258.9 ± 3.23^a^	20.4 ± 0.25^a^	44.6 ± 0.72^b^	7.26 ± 0.19^a^	5.70 ± 0.07^c^

Different letters in a column are significantly different at *p* < 0.05 according to Duncan’s multiple range test.

### System productivity, water use efficiency and production economics

Among the different combinations of CERM and winter crop treatments, ZTDSR_R+_ in rice-chickpea, rice-safflower and rice-lentil rotations led to the higher annual system productivity (15–39, 33–55 and 21–42%, respectively), system production efficiency (18–39, 11–31 and 23–41%, respectively), and water use efficiency (28–64, 45–100 and 40–76%, respectively) over ZTTPR and PTR treatments (Table 6). Treatment order for these parameters was ZTDSR_R+_ > ZTDSR > PTR_R+_ ≥ ZTTPR_R+_  ≥ PTR > ZTTPR (*p* < 0.05). Similarly, the highest net return was recorded in rice**-**safflower followed by rice**-**chickpea and rice**-**lentil rotations under ZTDSR_R+_ over the remaining CERM × winter crop treatment combinations (Table 7).

**Table 6:** System productivity and water productivity as influenced by different crop establishment methods and residues management (CERM) practices (2-year mean).

Parameter	CERM practice	Rice-chickpea	Rice-lentil	Rice- safflower	Rice-linseed	Rice-mustard	Mean		LSD (*p* = 0.05)
SREY (t ha^−1^)	ZTDSR	9.26 ± 0.32^ab^	9.07 ± 0.32^a^	8.90 ± 0.31^b^	7.34 ± 0.25^bc^	7.41 ± 0.28^ab^	8.40	CERM	0.46
	ZTDSR_R+_	9.97 ± 0.34^a^	9.69 ± 0.25^a^	9.79 ± 0.28^a^	8.54 ± 0.25^a^	7.81 ± 0.21^a^	9.16	WC	0.12
	ZTTPR	7.16 ± 0.31^d^	6.84 ± 0.29^d^	6.32 ± 0.22^d^	6.55 ± 0.31^d^	6.20 ± 0.34^c^	6.61	Interactions	*p* value
	ZTTPR _R+_	8.27 ± 0.41^c^	7.79 ± 0.30^bc^	7.37 ± 0.27^c^	7.44 ± 0.33^bc^	6.93 ± 0.37^bc^	7.56	Y × CERM	0.099
	PTR	7.86 ± 0.37^cd^	7.19 ± 0.29^cd^	6.47 ± 0.30^d^	6.94 ± 0.27^cd^	7.17 ± 0.34^ab^	7.13	Y × WC	< 0.0001
	PTR_R+_	8.64 ± 0.29^bc^	8.01 ± 0.29^b^	7.25 ± 0.28^c^	7.73 ± 0.34^b^	7.83 ± 0.40^a^	7.89	CERM × WC	< 0.0001
	Mean	8.53	8.10	7.68	7.42	7.23		Y × CERM × WC	0.114
SPE (kg ha^−1^ day^−1^)	ZTDSR	25.00 ± 0.90^b^	24.06 ± 0.83^b^	20.44 ± 0.67^bc^	20.26 ± 0.62^ab^	7.41 ± 0.28^ab^	19.43	CERM	1.21
	ZTDSR_R+_	27.36 ± 0.78^a^	26.40 ± 0.79^a^	23.43 ± 0.71^a^	21.60 ± 0.68^a^	7.81 ± 0.21^a^	21.32	WC	0.35
	ZTTPR	19.67 ± 0.99^d^	18.71 ± 0.87^d^	17.89 ± 0.90^d^	17.01 ± 0.80^c^	6.20 ± 0.34^c^	15.90	Interactions	*p* value
	ZTTPR _R+_	22.84 ± 1.01^bc^	21.44 ± 0.87^c^	19.98 ± 0.98^bc^	18.77 ± 0.86^bc^	6.93 ± 0.37^bc^	17.99	Y × CERM	0.089
	PTR	21.50 ± 0.87^cd^	19.32 ± 0.88^cd^	18.76 ± 0.78^cd^	19.38 ± 0.79^b^	7.17 ± 0.34^ab^	17.23	Y × WC	< 0.0001
	PTR _R+_	23.14 ± 1.01^bc^	21.07 ± 1.00^c^	21.03 ± 1.12^b^	21.87 ± 1.05^a^	7.83 ± 0.40^a^	18.99	CERM × WC	< 0.0001
	Mean	23.25	21.83	20.26	19.81	7.23		Y × CERM × WC	0.813
SWUE (kg ha^−1^ mm^−1^)	ZTDSR	5.78 ± 0.16^a^	5.63 ± 0.14^a^	5.58 ± 0.10^b^	4.62 ± 0.09^a^	4.62 ± 0.08^b^	5.25	CERM	0.24
	ZTDSR_R+_	6.07a ± 0.23^a^	5.92 ± 0.19^a^	6.02 ± 0.12^a^	5.22 ± 0.16^a^	4.83 ± 0.11^a^	5.61	WC	0.03
	ZTTPR	4.37 ± 0.18^bc^	4.03 ± 0.17^bc^	3.69 ± 0.13^c^	3.86 ± 0.16^bc^	3.70 ± 0.18^d^	3.93	Interactions	*p* value
	ZTTPR _R+_	4.73 ± 0.23^b^	4.24 ± 0.20^b^	4.15 ± 0.20^c^	4.09 ± 0.20^b^	3.87 ± 0.20^c^	4.22	Y × CERM	0.128
	PTR	3.71 ± 0.13^d^	3.36 ± 0.12^d^	3.01 ± 0.09^d^	3.23 ± 0.11^c^	3.30 ± 0.12^e^	3.32	Y × WC	< 0.0001
	PTR _R+_	3.87 ± 0.16^cd^	3.65 ± 0.16^d^	3.27 ± 0.11^cd^	3.64 ± 0.15^bc^	3.69 ± 0.18^e^	3.63	CERM × WC	< 0.0001
	Mean	4.75	4.47	4.29	4.11	4.00		Y × CERM × WC	< 0.0001

*SREY* system rice equivalent yield, *SPE* system production efficiency, *SWUE* system water use efficiency, *WC* winter crop, *Y* year.

Different letters in a column are significantly different at *p* < 0.05 according to Duncan’s multiple range test.

**Table 7:** Production economics as influenced by crop establishment methods and residues management (CERM) practices (2-year mean).

Parameter	CERM practice	Rice-chickpea	Rice-lentil	Rice- safflower	Rice-linseed	Rice-mustard	Mean		LSD (*p* = 0.05)
SGR (× 10^3^ INR ha^−1^)	ZTDSR	164.2 ± 8.18^ab^	151.3 ± 6.31^ab^	153.5 ± 5.92^b^	127.5 ± 4.32^bc^	130.7 ± 4.09^ab^	145.4	CERM	11.6
	ZTDSR_R+_	183.5 ± 6.37^a^	169.0 ± 6.08^a^	175.4 ± 4.05^a^	150.4 ± 5.06^a^	144.3 ± 4.04^a^	164.5	WC	1.74
	ZTTPR	137.8 ± 5.80^c^	124.0 ± 5.02^d^	115.4 ± 3.98^d^	120.1 ± 5.34^c^	116.2 ± 5.80^b^	122.7	Interactions	*p* value
	ZTTPR _R+_	149.2 ± 8.90^bc^	134.0 ± 7.78^bcd^	132.8 ± 6.65^c^	126.4 ± 7.22^bc^	122.0 ± 7.00^b^	132.9	Y × CERM	0.605
	PTR	148.6 ± 7.11^bc^	130.9 ± 5.41^cd^	122.4 ± 4.67^cd^	127.7 ± 5.74^bc^	132.7 ± 5.84^ab^	132.4	Y × WC	< 0.0001
	PTR _R+_	155.1 ± 7.52^bc^	143.7 ± 6.89^bc^	130.1 ± 5.99^cd^	142.4 ± 6.95^ab^	142.0 ± 6.97^a^	142.7	CERM × WC	< 0.0001
	Mean	156.4	142.2	138.3	132.4	131.3		Y × CERM × WC	0.134
SNR (× 10^3^ INR ha^−1^)	ZTDSR	101.0 ± 5.39^ab^	96.7 ± 4.83^a^	100.3 ± 4.02^b^	74.4 ± 2.74^b^	75.5 ± 2.98^ab^	89.6	CERM	6.20
	ZTDSR_R+_	117.8 ± 7.40_a_	110.7 ± 5.58^a^	120.0 ± 3.97^a^	93.0 ± 4.46^a^	86.0 ± 3.16^a^	105.5	WC	1.18
	ZTTPR	68.7 ± 6.06^c^	62.1 ± 4.81^b^	55.9 ± 3.40^d^	56.8 ± 4.73^c^	52.8 ± 5.25^c^	59.3	Interactions	*p* value
	ZTTPR _R+_	78.2 ± 7.90^c^	69.6 ± 6.69^b^	71.0 ± 6.41^c^	64.2 ± 5.75^bc^	61.3 ± 5.83^bc^	68.9	Y × CERM	0.072
	PTR	78.3 ± 5.91^c^	70.2 ± 5.04^b^	62.1 ± 3.91^cd^	65.8 ± 4.56^bc^	71.5 ± 4.99^ab^	69.6	Y × WC	< 0.0001
	PTR _R+_	84.9 ± 6.90^bc^	78.7 ± 6.47^b^	68.6 ± 4.88^cd^	78.9 ± 6.11^ab^	82.6 ± 7.00^a^	78.7	CERM × WC	< 0.0001
	Mean	88.2	81.4	79.7	72.2	71.6		Y × CERM × WC	0.0014
SBCR	ZTDSR	2.49 ± 0.10^a^	2.56 ± 0.10^b^	2.75 ± 0.10^b^	2.28b ± 0.09	2.37 ± 0.09^ab^	2.49	CERM	0.18
	ZTDSR_R+_	2.76 ± 0.12^a^	2.87 ± 0.10^a^	3.18 ± 0.08^a^	2.73 ± 0.10^a^	2.63 ± 0.07^a^	2.84	WC	0.02
	ZTTPR	1.84 ± 0.10^b^	1.85 ± 0.09^d^	1.83 ± 0.08^d^	1.89 ± 0.10^c^	1.83 ± 0.11^d^	1.85	Interactions	*p* value
	ZTTPR _R+_	2.07 ± 0.11^b^	2.01 ± 0.10^cd^	2.13 ± 0.10^c^	2.06 ± 0.10^bc^	2.01 ± 0.11^cd^	2.05	Y × CERM	0.600
	PTR	2.07 ± 0.08^b^	2.04 ± 0.08^cd^	1.99 ± 0.08^cd^	2.07 ± 0.09^bc^	2.16 ± 0.10^bc^	2.07	Y × WC	< 0.0001
	PTR _R+_	2.15 ± 0.11^b^	2.17 ± 0.09^c^	2.10 ± 0.09^c^	2.24 ± 0.12^b^	2.35 ± 0.12^ab^	2.20	CERM × WC	< 0.0001
	Mean	2.23	2.25	2.33	2.21	2.23		Y × CERM × WC	0.0002

*SGR* system gross return, *SNR* system net return, *SBCR* system benefit cost ratio, *WC* winter crop, *Y* Year.

Different letters in a column are significantly different at *p* < 0.05 according to Duncan’s multiple range test.

### Soil cracks and soil moisture dynamics

**Figure 2:**
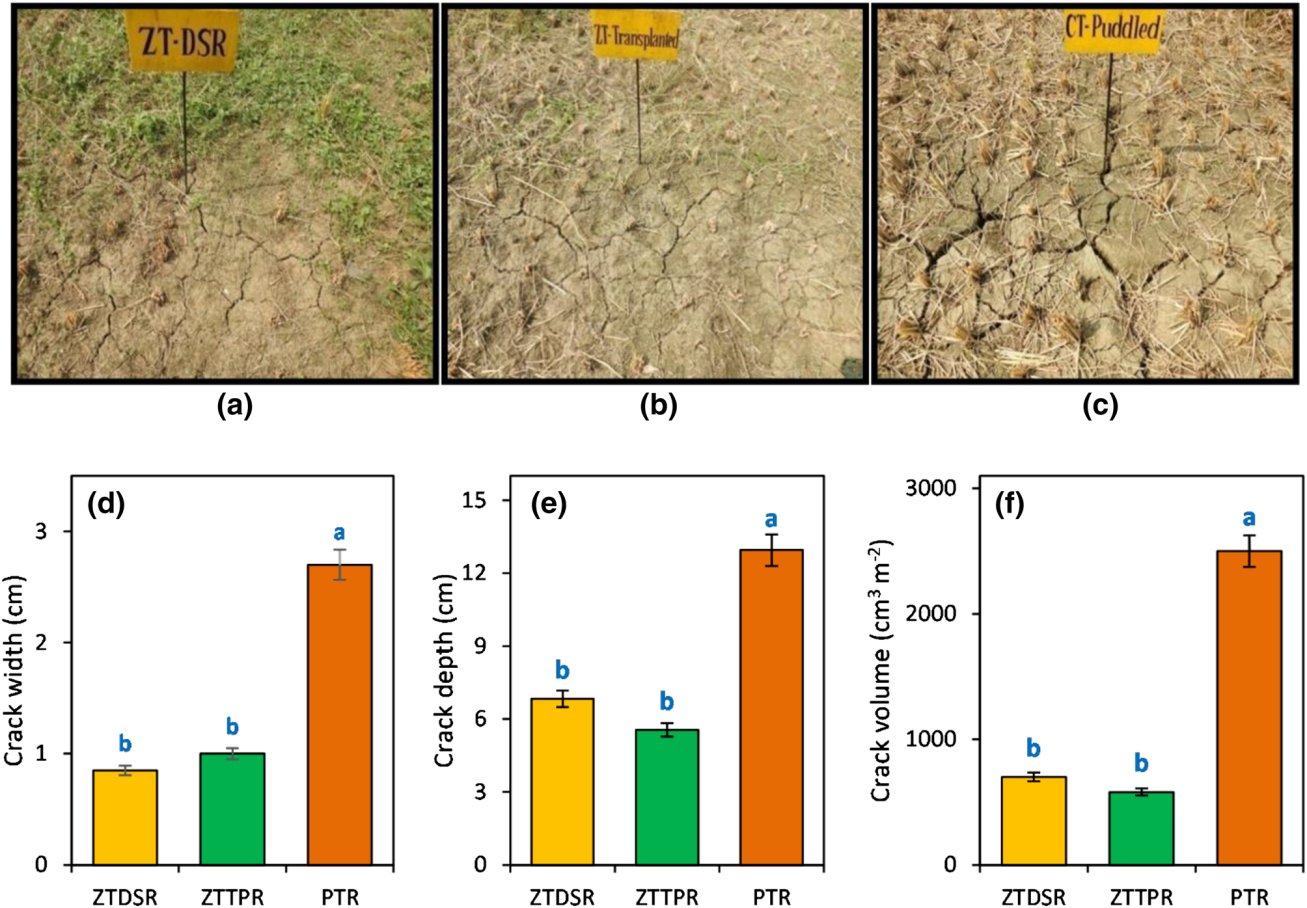
Field view of soil cracks under different tillage cum crop establishment practices (**a**–**c**). Crack width (**d**), depth (**e**) and volume (**f**) as influenced by different tillage cum crop establishment practices (2-year mean). Error bar represents the standard error of mean; Different lowercase letters correspond to treatments are significantly different at *p* < 0.05 by Duncan’s multiple range test.

**Figure 3:**
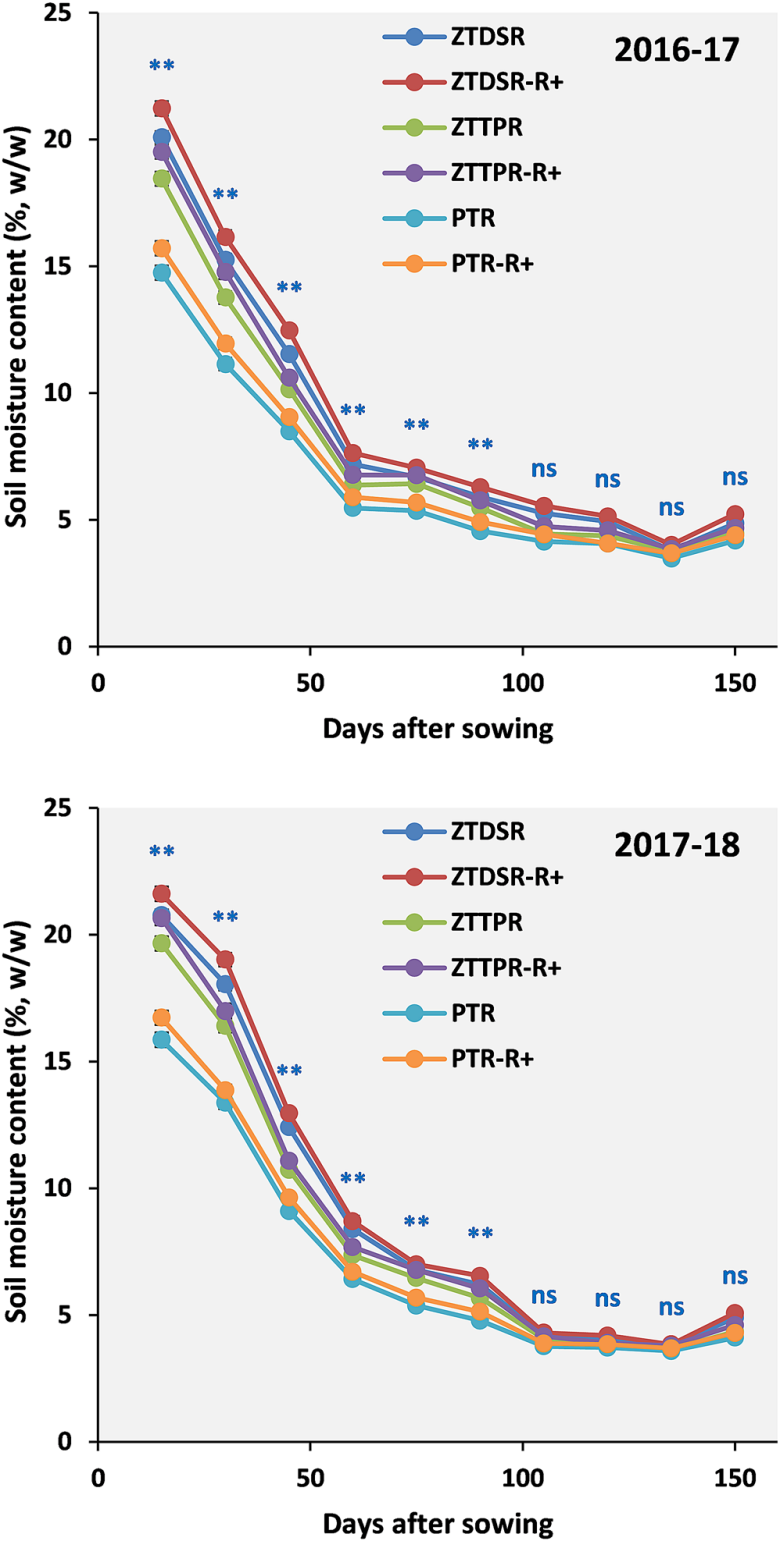
Soil moisture content (w/w) under different tillage and residue management treatments during 2016–2017 and 2017–2018; *significantly different at *p* < 0.05; ‘*ns*’ non-significant.

**Figure 4:**
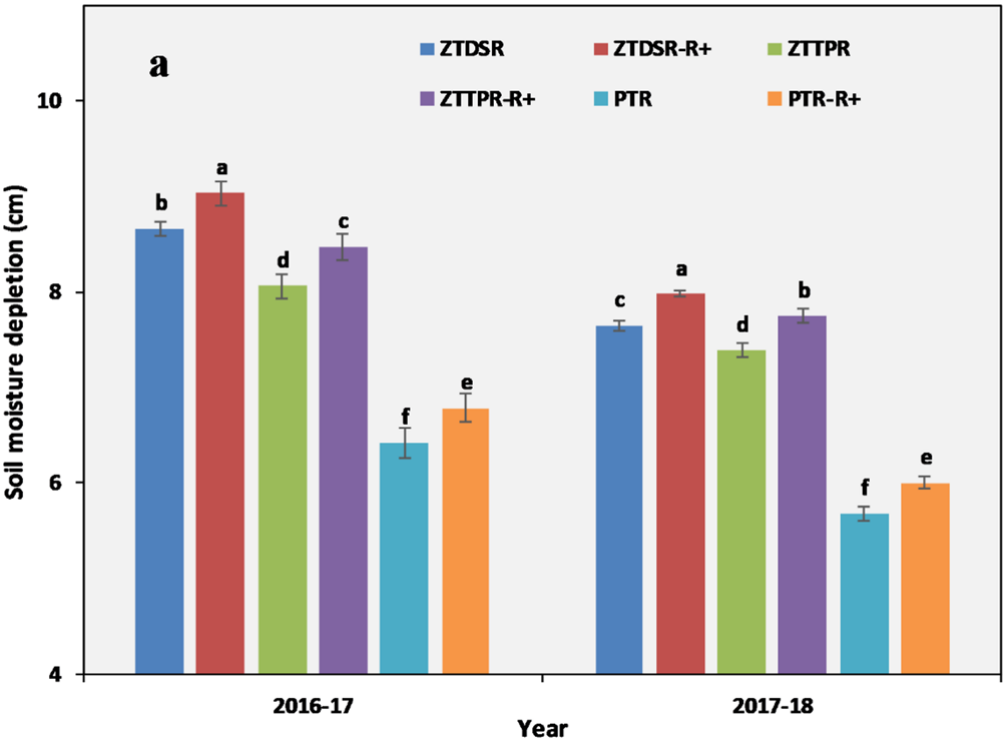
Soil moisture depletion from soil profile (0*–*30 cm) under different tillage and residue management practices. Error bar indicates standard error of mean. Different lowercase letters correspond to treatments are significantly different at *p* < 0.05 by Duncan’s multiple range test.

Higher crack width was observed in PTR treatment and reduced in ZTDSR and ZTTPR treatments (Fig. 2). Crack depth was reduced in ZTTPR (57%, *p* < 0.05) and ZTDSR (47%, *p* < 0.05) treatments compared to PTR treatment. Crack volume was markedly higher (2.6–3.3 times) in PTR treatment. Irrespective of CERM practices, soil moisture depletion was higher up to 60 DAS (Fig. 2). During the initial growth stages, ZTDSR_R+_ treatment maintained 35 and 43% higher soil moisture (*p* < 0.05) over ZTTPR_R+_ and PTR_R+_ treatments, respectively (2016–2017) (Fig. 3). A similar treatment trend for soil moisture content was observed in the second year (2017–2018), and ZTDSR_R+_ treatment retained 36% and 24% higher soil moisture over PTR treatment at the time of sowing and harvesting stages, respectively. Soil moisture depletion and crop water use efficiency followed the trend of ZTDSR > ZTTPR > PTR treatments (Fig. 4).

### Energy budgeting and energy use efficiency

Crop residues accounted for a major share of energy input that influenced the system energy output and energy use efficiency (Table 8). Higher input energy was recorded in PTR_R+_ treatment over conservation tillage treatment (ZTDSR). The system net energy output was higher in residue removal treatment and was higher under ZTDSR for rice–safflower followed by rice**-**lentil and rice**-**chickpea rotations. A similar result was observed for energy ratio also.

**Table 8:** Energy budgeting and energy ratio as influenced by crop establishment methods and residues management (CERM) practices (2-year mean).

Parameter	CERM practice	Rice-chickpea	Rice-lentil	Rice- safflower	Rice-linseed	Rice-mustard	Mean		LSD (*p* = 0.05)
SEI (× 10^3^ MJ ha^−1^)	ZTDSR	23.1^f^	22.5^f^	22.5^f^	24.1^f^	23.1^f^	23.0	CERM	–
	ZTDSR_R+_	68.1^b^	67.5^b^	67.5^b^	69.1^b^	68.1^b^	68.0	WC	–
	ZTTPR	25.8^e^	25.1^e^	25.1^e^	26.7^e^	25.8^e^	25.7	Interactions	*p* value
	ZTTPR _R+_	65.8^c^	65.1^c^	65.1^c^	66.7^c^	65.8^c^	65.7	Y × CERM	0.6544
	PTR	28.9^d^	28.2^d^	28.2^d^	29.8^d^	28.9^d^	28.8	Y × WC	–
	PTR _R+_	80.1^a^	79.5^a^	79.5^a^	81.1^a^	80.1^a^	80.1	CERM × WC	–
	Mean	48.6	48.0	48.0	49.6	48.6		Y × CERM × WC	–
SEO (× 10^3^ MJ ha^−1^)	ZTDSR	203.6 ± 5.8^b^	207.9 ± 5.1^b^	233.7 ± 6.6^b^	187.3 ± 4.1^b^	197.8 ± 3.2^b^	206.1	CERM	15,023
	ZTDSR_R+_	229.4 ± 6.5^a^	231.0 ± 5.0^a^	262.1 ± 6.0^a^	215.8 ± 4.3^a^	221.4 ± 4.9^a^	231.9	WC	2063
	ZTTPR	178.4 ± 6.5^c^	177.5 ± 4.8^c^	180.7 ± 5.2^d^	172.8 ± 4.7^c^	180.6 ± 4.6^c^	178.0	Interactions	*p* value
	ZTTPR _R+_	182.1 ± 5.3^c^	181.3 ± 4.9^c^	194.9 ± 6.1^d^	177.5 ± 4.8^bc^	186.0 ± 5.1^bc^	184.4	Y × CERM	0.6258
	PTR	217.5 ± 5.9^ab^	218.2 ± 5.6^ab^	214.9 ± 6.0^c^	205.5 ± 5.0^a^	218.5 ± 6.7^a^	215.0	Y × WC	0.0872
	PTR _R+_	219.7 ± 5.1^a^	218.9 ± 3.6^ab^	215.1 ± 4.1^c^	216.7 ± 4.4^a^	228.6 ± 5.0^a^	219.8	CERM × WC	< 0.0001
	Mean	205.1	205.8	216.9	196.0	205.5		Y × CERM × WC	0.3529
SNEO (× 10^3^ MJ ha^−1^)	ZTDSR	180.4 ± 5.8^a^	185.4 ± 5.1^a^	211.2 ± 6.6^a^	163.2 ± 4.1^a^	174.7 ± 3.2^a^	183.0	CERM	15,023
	ZTDSR_R+_	161.2 ± 6.5^b^	163.5 ± 5.0^b^	194.6 ± 6.0^b^	146.8 ± 4.3^b^	153.3 ± 4.9^b^	163.9	WC	2063
	ZTTPR	152.6 ± 6.5^bc^	152.4 ± 4.8^bc^	155.6 ± 5.2^c^	146.1 ± 4.7^b^	154.8 ± 4.6^b^	152.3	Interactions	*p* value
	ZTTPR _R+_	116.3 ± 5.3^d^	116.2 ± 4.9^d^	129.7 ± 6.1^d^	110.8 ± 4.8^c^	120.2 ± 5.1^c^	118.7	Y × CERM	0.6258
	PTR	188.6 ± 5.9^a^	190.0 ± 5.6^a^	186.7 ± 6.0^b^	175.7 ± 5.0^a^	189.6 ± 6.7^a^	186.1	Y × WC	0.0872
	PTR _R+_	139.6 ± 5.1^c^	139.4 ± 3.6^c^	135.6 ± 4.1^d^	135.6 ± 4.4^b^	148.5 ± 5.0^b^	139.8	CERM × WC	< 0.0001
	Mean	156.5	157.8	168.9	146.4	156.9		Y × CERM × WC	0.3529
SER	ZTDSR	8.81 ± 0.25^a^	9.26 ± 0.23^a^	10.39 ± 0.29^a^	7.78 ± 0.17^a^	8.56 ± 0.14^a^	8.96	CERM	0.42
	ZTDSR_R+_	3.37 ± 0.10^d^	3.42 ± 0.07^d^	3.88 ± 0.09^c^	3.13 ± 0.06^d^	3.25 ± 0.07^d^	3.41	WC	0.07
	ZTTPR	6.92 ± 0.25^c^	7.07 ± 0.19^c^	7.19 ± 0.21^b^	6.47 ± 0.18^c^	7.01 ± 0.18^c^	6.93	Interactions	*p* value
	ZTTPR _R+_	2.77 ± 0.08^e^	2.78 ± 0.08^e^	2.99 ± 0.09^d^	2.66 ± 0.07^e^	2.83 ± 0.08^e^	2.81	Y × CERM	0.4201
	PTR	7.53 ± 0.20^b^	7.74 ± 0.20^b^	7.61 ± 0.21^b^	6.89 ± 0.17^b^	7.57 ± 0.23^b^	7.47	Y × WC	0.1704
	PTR _R+_	2.74 ± 0.06^e^	2.75 ± 0.05^e^	2.71 ± 0.05^d^	2.67 ± 0.05^e^	2.85d ± 0.06^e^	2.75	CERM × WC	< 0.0001
	Mean	5.36	5.50	5.79	4.93	5.35		Y × CERM × WC	0.5645

*SEI* system energy input, *SEO* system energy output, *SNEO* system net energy output, *SER* system energy ratio, *WC* winter crops, *Y* year.

Different letters in a column are significantly different at *p* < 0.05 according to Duncan’s multiple range test.

### Emission of greenhouse gases (GHGs) and global warming potential (GWP)

**Figure 5:**
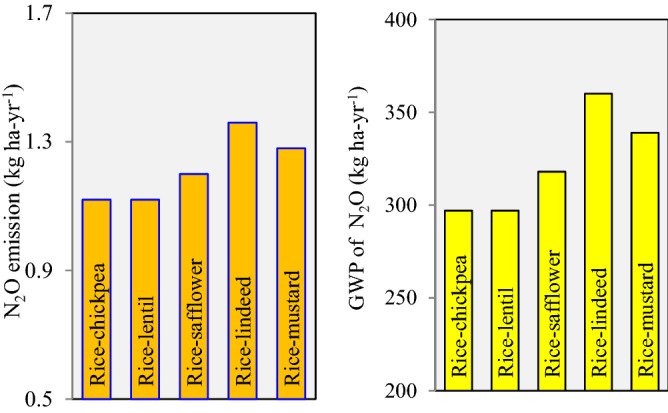
Emission of N_2_O and global warming potential (GWP) of N_2_O as influenced by different crop rotations in rice-fallow (2-year mean).

Simulation results revealed that ZTDSR led to the minimum emission of GHGs and GWP compared to ZTTPR and PTR treatments (Fig. 5, Table 9). ZTTPR and PTR treatments had a higher GWP than that to ZTDSR*.* Among the cropping system, legume inclusive rotations i.e. rice**-**chickpea and rice**-**lentil had lower N_2_O emission compared to oilseed inclusive rotation (rice**-**safflower, rice**-**linseed and rice**-**mustard). However, the highest (0.093 kg CO_2_ eq kg^−1^ SREY) GHGI was recorded in rice**-**linseed and rice-safflower rotations and the lowest (0.076 kg CO_2_ eq kg^−1^ SREY) with the rice-chickpea system.

**Table 9:** Global warming potential (GWP) and greenhouse gas intensity (GHGI) as influenced by crop establishment methods and residues management (CERM) practices and winter crops (2-year mean).

Parameter	CERM practice	Rice-chickpea	Rice-lentil	Rice-safflower	Rice-linseed	Rice-mustard	Mean
GWP of CH_4_ (kg ha yr^−1^)	ZTDSR	157^b^	157^b^	157^a^	157^a^	157^a^	157
	ZTDSRR +	157^b^	157^b^	157^a^	157^a^	157^a^	157
	ZTTPR	358^a^	358^a^	358^b^	358^b^	358^b^	358
	ZTTPR R +	358^a^	358^a^	358^b^	358^b^	358^b^	358
	PTR	358^a^	358^a^	358^b^	358^b^	358^b^	358
	PTR R +	358^a^	358^a^	358^b^	358^b^	358^b^	358
System GWP (kg ha yr^−1^)	ZTDSR	454^b^	454^b^	475^b^	517^b^	496^b^	479
	ZTDSRR +	454^b^	454^b^	475^b^	517^b^	496^b^	479
	ZTTPR	655^a^	655^a^	676^a^	719^a^	698^a^	681
	ZTTPR R +	655^a^	655^a^	676^a^	719^a^	698^a^	681
	PTR	655^a^	655^a^	676^a^	719^a^	698^a^	681
	PTR R +	655^a^	655^a^	676^a^	719^a^	698^a^	681
	Mean	588	588	609	652	631	
GHGI (kg CO_2_ eq kg^−1^ SREY)	ZTDSR	0.049^d^	0.050^c^	0.053^c^	0.070^d^	0.067^d^	0.058
	ZTDSRR +	0.045^d^	0.047^c^	0.048^c^	0.061^e^	0.064^d^	0.053
	ZTTPR	0.092^a^	0.096^a^	0.107^a^	0.110^a^	0.113^a^	0.104
	ZTTPR R +	0.079^bc^	0.084^b^	0.092^bc^	0.097^bc^	0.101^b^	0.091
	PTR	0.083^b^	0.091^a^	0.105^a^	0.104^ab^	0.097_b_	0.096
	PTR R +	0.076^c^	0.082^b^	0.093^b^	0.093^c^	0.089^c^	0.087
	Mean	0.076	0.082	0.093	0.093	0.089	

*GWP* global warming potential, *GHGI* greenhouse gas intensity.

Different letters in a column are significantly different at *p* < 0.05 according to Duncan’s multiple range test.

### Principal component analysis (PCA)

**Figure 6:**
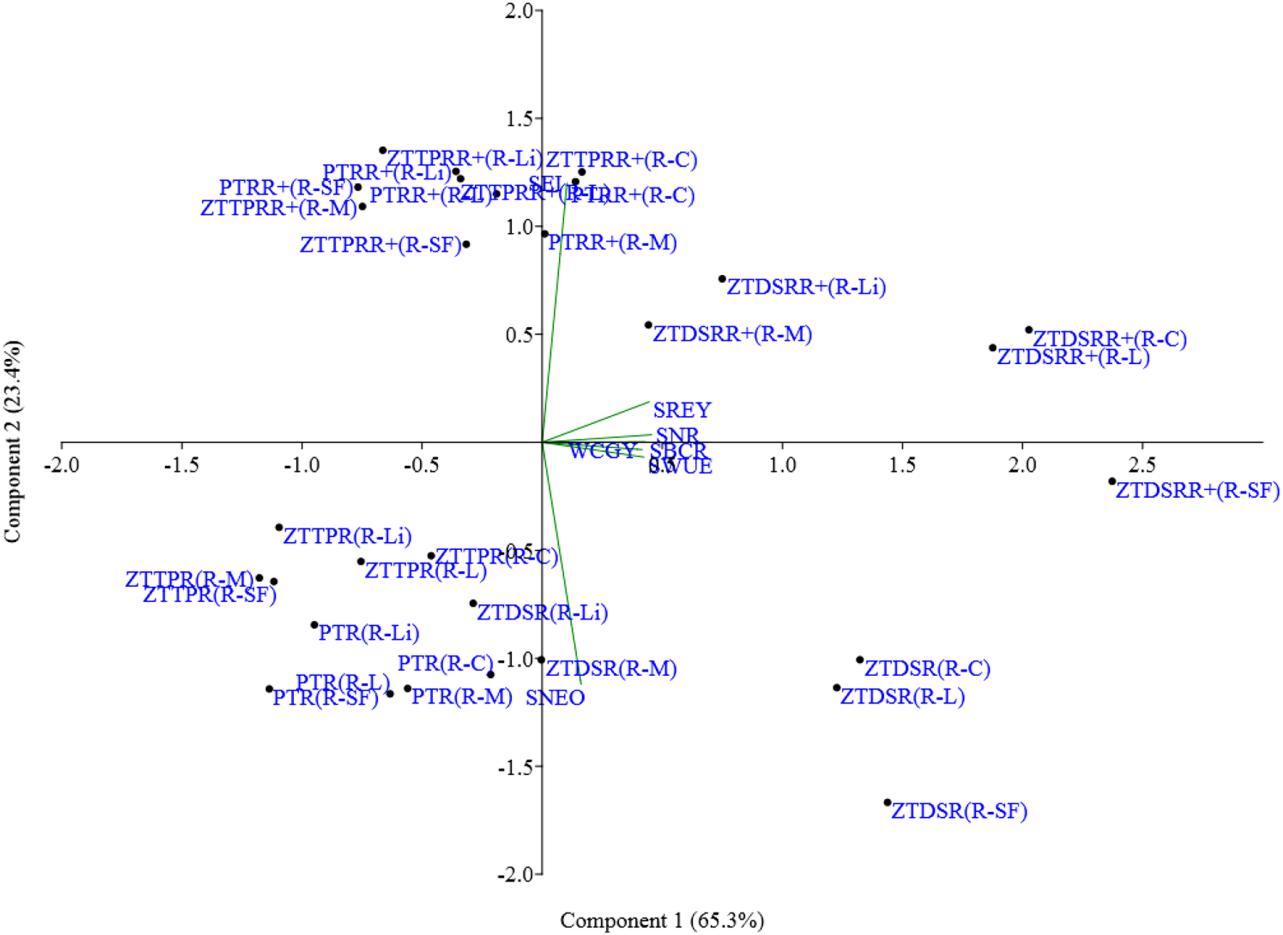
Scatter plot of treatments [CERM × WC] on PCA coordinates. R-C: rice-chickpea; R-L: rice-lentil; R-SF rice-safflower; R-Li: rice-linseed; R-M: rice-mustard. *SREY*: system rice equivalent yield; *WCGY:* winter crop grain yield, SNR: system net returns; *SBCR:* system benefit cost ratio; *SEI:* system energy input; *SNEO:* system net energy output; *SWUE:* system water use efficiency.

The PCA result showed that rice**-**chickpea, rice**-**lentil, and rice**-**safflower rotations with ZTDSR/ZTDSR_R+_ had a higher weightage of principal component 1, which represented 65.3% of total variability, and distinctly located on PCA coordinates apart from other CERM × crop rotation treatments (Fig. 6). Results also showed that parameters SREY, system benefit–cost ratio, system net return, system water use efficiency and winter crop yield had a close association with each other.

## Discussion

### Crop productivity

Despite multipronged challenges in the rice fallow agro-ecosystem, there is an extensive scope to grow a post-rainy season crop through appropriate soil moisture conservation and bio-system engineering approaches^16^. Strategic choice of the winter crop(s) for rice-fallow areas is crucial for maximizing the system productivity and farmers' income^6^. Soil characteristics and hydrology of rice**-**fallow areas differ considerably^9, 12^. Rice**-**fallows of eastern India are typically lowland having compacted sub-surface soil due to puddling in the rice crop season. Our results showed that grain legumes like chickpea and lentil have higher yield potential under the water-limited rice-fallow condition. Certainly, root traits had oversized importance for efficient use of residual soil moisture and nutrients under water-limited rice-fallows conditions^24^. Deep-rooted grain legumes have higher capacity to utilize the soil moisture from the lower soil depths^13^ Furthermore, nutrient management in post-rainy season crop is challenging due to moisture stress at later growth stages, particularly in oilseed crops, which demands split application of fertilizer nitrogen for realizing potential productivity^11, 16^. Grain legumes are less dependent of fertilizer nitrogen and that may a reason for higher yield over oilseed crops. Among the oilseed crops, safflower performed well in terms of crop productivity in rice fallows indicating that crop has potential to withstand water-deficit stress in rice-fallows.

Where, the low plant stands (data not presented) and forced maturity of mustard and linseed crops due to higher ambient temperatures during the later growth stages adversely affected the crop yield. Results demonstrated that conservation tillage with crop residue retention could increase the yield of winter crops in rice-fallows by improving soil physical properties and moisture retention. Lower length, width and volume of cracks under conservation tillage indicate better soil aggregation and pores size distribution^10, 12, 16, 25^. Several studies have reported the adverse impact of conventional PTR cultivation on soil health and microbial functions that in turn affect the successive winter crop performance^25, 26^. Formation of hard layer in the sub-surface soil due to wet-tillage increases the soil resistance that restricts the root growth of successive crop and limits water extraction from lower soil depths^27^. Bandyopadhyay et al*.*^28^ reported that puddled transplanted rice results in frequent drying and develops deep cracks causing losses of soil moisture and affects winter crop productivity. Our result also showed that wet-tillage in PTR increased the soil cracking that possibly a factor of increased loss of soil moisture. Alternative tillage and crop establishment methods in rice should be aimed at fewer inputs to produce at par crop yields compared with PTR production system^29, 30^. Rice grain yield was lower under conservation tillage treatments compared to PTR treatment. This might be due to higher crop-weed competition under conservation tillage practices has also been reported to be one of yield limiting factors^16^. The yield advantages of conservation tillage practices in rice are mostly evident in long-run with the increase in water stable aggregates, soil organic carbon, and increased availability of soil nutrients^26, 31^. Higher system productivity was associated with grain legumes (chickpea and lentil) and safflower inclusive crop rotations under ZTDSR_R+_ treatment due to higher yields of the respective crops. The PCA analysis also confirm that system rice equivalent yield (SREY), system net return (SNR) and system benefit–cost ratio (SBCR) were positively influenced with ZTDSR_R +_ (R-C), ZTDSR_R +_ (R-L), ZTDSR_R+_ (R-SF) production system. It indicates that growing of chickpea, lentil and safflower under the conservation agriculture production system is a better option for the farming community of rice fallow areas in eastern India^19^.

### Soil moisture dynamics and water use efficiency

Soil moisture deficit is the major abiotic stress factor that limits the winter crop productivity in rice**-**fallows. During the investigation, a negligible amount of rainfall was received during the winter season, while the evaporative loss was much higher, resulted in fast depletion of the soil moisture from the root zone (0–30 cm)^16^. The conservation tillage practices treatment (particularly ZTDSR) was effective in conserving soil moisture in upper soil depths and maintained a higher level of moisture for an extended period. Fundamentally, soil hydrology is largely influenced by the tillage practices^35^. Soil moisture distribution in the undisturbed soil profile is moderated by capillary continuity and crop residue retention further minimizes the evaporative loss^36^. The higher ambient temperature at later stages of winter crops (100 DAS onwards) increased the evaporative loss of soil moisture and thus the effect of CERM practices on soil moisture was marginal at later growth stages of crop. Several studies have reported that standing stubbles in CA practices could reduce the soil evaporative losses of moisture and moderates the soil temperature by shading effects^32–34, 37^.

### Production economics

Production economics is the primary driver of cropping intensification in rice-fallows areas, where the farmers are mostly poor with marginal land holdings^6, 7, 11, 16^. The higher profit margin in conservation tillage practices with rice residue retention was primarily attributed to a reduction in tillage and irrigation costs in rice and increased return from winter crops, which surpassed the marginal reduction in rice yield compared to conventional practice (PTR_R+_). Equally, production potential and market price of winter crops directly influenced the profit margin. The result suggests that rice residue retention could be an economically viable and sustainable crop management option for rice-fallows cropping intensification. The economic advantage of conservation tillage practices in rice-based systems has also been reported earlier from non-rice fallow production systems ^31–33^.

### Energy budgeting and energy use efficiency

Improved energy productivity remains crucial for sustainable crop production in long-run^38, 39^. Nowadays, the sustainability of rice-based production systems is questioned due to excessive use of energy sources, degradation of natural resources (groundwater and soil), declining factor productivity and profit margin^40^. Our results showed that the strategic choice of crop rotations and conservation tillage practices could minimize energy inflow and increase energy ratio over energy intensive PTR production system. Rice and winter crop production, tillage and irrigation were the major variables of energy. Rice residue retention markedly reduced the energy ratio as it largely increased the bio-energy input. Nevertheless, crop residue retention has been suggested as a sustainable approach particularly in the tropical agro-climate, where the soil native carbon pool is low^25, 26^. Further, residue retention could be the best alternative to crop residue burning, which is extensively being practiced in rice-growing areas of Indo-Gangetic plains.

### Greenhouse gases (GHGs) and global warming potential (GWP)

Conventional PTR production system, which is highly labour intensive, can effectively be substituted by ZTDSR with a little negative impact on rice grain yield but more importantly with a significant reduction in water use, CH_4_ emissions and higher net profits. The GWP is steered by cultivar-environment-management interactions and, thus developing a high-yielding rice variety adapted to an improved CERM option with low GWP should be a future priority ^41, 42^. Thus, legume-inclusive rotations are better to rationalize the energy and GWP due to lower input requirements. Among the farm operations that can lower GHG emissions include minimum use of nitrogen fertilizer, inclusion of pulses and adoption of ZTDSR for rice crop establishment in rice-fallows. The GWP can be lowered by adopting CA production system, maximizing biomass production, improving input use efficiency, decreasing emission of GHGs (CH_4_ and N_2_O) and building up of soil organic carbon^43^. With a lower requirement of nitrogenous fertilizer and irrigations, legume-based cropping systems in rice-fallows have a strong potential to reduce energy use and GWP, while maintaining similar net returns as those from other crop rotations^40^. Thus, these rotations may be preferred options towards the sustainable cleaner, safer agricultural production system under the scarce resources in the rice-fallow areas of South Asia.

## Conclusion

Our study concluded that chickpea, lentil and safflower could be the candidate crops for sustainable cropping intensification of rice-fallow areas in eastern India. Grain legumes along with safflower inclusive rotations led to higher system productivity, energy productivity and economic returns in rice-fallows conditions. Moreover, conservation tillage practices with rice residue retention (~ 30%), (particularly ZTDSR_R+_) markedly improved the winter crop productivity due to higher soil moisture retention over PTR production system. The relative responses of winter crops to conservation tillage and residue retention practices were better for safflower followed by chickpea and lentil. Thus, the study suggests that inclusion of grain legumes (chickpea and lentil) and safflower with conservation tillage and rice residue retention could be a sustainable approach for cropping intensification in rice-fallow areas for food, nutritional and environmental security. Systemic future research on soil moisture conservation and nutrient management, cultivars selection, and farm mechanization is needed that may further upscale the productivity and profitability of rice-fallows agro-ecosystem in eastern India.

## Supplementary information

Supplementary file1 (DOCX 27 kb)

## References

[1] Kumar R, Mishra JS, Upadhyay PK, Hans H (2019). Rice fallows in the Eastern India: problems and prospects. Indian J. Agric. Sci..

[2] Gumma MK (2016). Mapping of rice–fallow crop land areas for short-season grain legumes intensification in South Asia using MODIS 250 m time-series data. Int. J. Dig. Earth.

[3] Layek J, Chowdhury S, Ramkrushna GI, Das A (2014). Evaluation of different lentil cultivars in low land rice-fallow under no-till system for enhancing cropping intensity and productivity. Indian J. Hill Farm..

[4] Yadav GS, Datta M, Saha P, Debbarma C (2015). Evaluation of lentil varieties/lines for utilization of rice fallow in Tripura. Indian J. Hill Farm..

[5] Singh RG (2012). Opportunities for managing rice fallow systems with conservation agriculture technologies. Indian Farm..

[6] Ghosh PK, Hazra KK, Nath CP, Das A, Acharya CL (2016). Scope, constraints and challenges of intensifying rice (*Oryza sativa* L.) fallows through pulses. Indian J. Agron..

[7] Mishra, J.S., Kumar, R. & Bhatt, B.P. Low**-**cost technologies for management of rice fallows in Eastern India. in* XXI Biennial National Symposium of Indian Soc. Agron.*, 24–26 Oct. 2018. MAPUAT, Udaipur, Rajasthan, India. 7**–**9 (2018).

[8] Kumar R, Mishra JS, Kumar R (2018). Enhancing the productivity of rice fallows area of Eastern India through inclusion of pulses and oilseeds. Indian Farm..

[9] Bandyopadhyay KK (2015). Characterization and crop planning of rabi-fallows using remote sensing and GIS. Curr. Sci..

[10] Singh RN (2017). Influence of rice (*Oryza sativa* L.) habit groups and moisture conservation practices on soil physical and microbial properties in rice + lathyrus relay cropping system under rice fallows in Eastern Plateau of India. Indian J. Agric. Sci..

[11] Kumar, R. et al*.* Evaluation of crop establishment techniques in rice-fallows of Eastern Indo**-**Gangetic Plains. in* National Conference on Organic Waste management for Food and Environmental Security under theme Theme Crop Residue Management*, 8–10 Feb., 2018. ICAR**-**Indian Institute of Soil Science Bhopal, Madhya Pradesh, India (2018b).

[12] Bandyopadhyay PK (2016). Effects of stubble length of rice in mitigating soil moisture stress and on yield of lentil (*Lens culinaris Medik*) in rice*-*lentil relay crop. Agric. Water Manage..

[13] Hazra KK, Bohra A (2020). Increasing relevance of pulse crops to sustainable intensification of Indian agriculture. Nat. Acad. Sci. Lett..

[14] Verhulst N (2011). Wheat yield and tillage**-**straw management system × year interaction explained by climatic co**-**variables for an irrigated bed planting system in north**-**western Mexico. Field Crops Res..

[15] Sidhu, H. S. et al. Development of happy seeder for direct drilling into combine harvested rice. in *Permanent bed and rice residue management for Rice-Wheat systems in Indo-Gangetic Plains Proceedings of a workshop* (Humphreys, E., Roth, C Eds.), 7**–**9 Sept.2006, Ludhiana, Punjab, India. ACIAR No. **127**, pp. 159**–**170 (2008).

[16] Kumar R, Mishra JS, Rao KK (2019). Sustainable intensification of rice fallows of Eastern India with suitable winter crop and appropriate crop establishment technique. Environ. Sci. Pollut. Res..

[17] Richards LA (1949). Methods for measuring soil moisture tension. Soil Sci..

[18] Dasog GS, Shashidhara GB (1993). Dimension and volume of cracks under different crop covers. Soil Sci..

[19] Dasog GS, Acton DI, Mermut AR, de Jong E (1988). Shrink**-**swell potential and cracking in clay soils of Saskatchewan. Can. J. Soil Sci..

[20] Devasenapathy P, Senthilkumar G, Shanmugam PM (2009). Energy management in crop production. Indian J. Agron..

[21] IPCC. Climate change 2013 the physical science basis. Contribution of Working Group I to Fifth Assessment Report of Inter-Governmental Panel on Climate Change (*Eds.* Stocker, T.F. et al.) 710**–**716 (Cambridge Uni. Press, Cambridge and New York, 2013).

[22] Padre TA (2016). Quantifying changes to the global warming potential of rice**-**wheat systems with adoption of conservation agriculture in north**-**western India. Agric. Ecosyst. Environ..

[23] Pratibha G (2015). Impact of conservation agriculture practices on energy use efficiency and global warming potential in rainfed pigeonpea**-**castor systems. Euro. J. Agron..

[24] Mishra, J. S. et al. Evaluation of pulses and oilseed under different crop establishment methods in rice**-**fallows of Eastern India. Paper ID No. IAC**-**2016/Sym**-**.XII/107). in* 4th International Agronomy Congress on “Agronomy for Sustainable Management of Natural Resources, Environment, Energy and Livelihood Security to Achieve Zero Hunger Challenge”*, 22**–**26 Nov., 2016, New Delhi, India (2016).

[25] Das A (2018). Tillage and cropping sequence effect on physico**-**chemical and biological properties of the soil in Eastern Himalayas. India. Soil Till. Res..

[26] Nandan N (2019). Impact of conservation tillage in rice**-**based cropping systems on soil aggregation, carbon pools and nutrients. Geoderma.

[27] Kar G, Kumar A (2009). Evaluation of post**-**rainy season crops with residual soil moisture and different tillage methods in rice fallow of eastern India. Agric. Water Manage..

[28] Bandyopadhyay PK (2018). Response of lentil (*Lens culinaris*) to post-rice residual soil moisture under contrasting tillage practices. Agric. Res..

[29] Jat HS, Sharma PC, Datta A, Choudhary M, Kakraliya SK, Singh Y, Sidhu HS, Gerard B, Jat ML (2019). Re-designing irrigated intensive cereal systems through bundling precision agronomic innovations for transitioning towards agricultural sustainability in North-West India. Sci. Rep..

[30] Chakraborty D, Ladha JK, Rana DS, Jat ML, Gathala MK, Yadav S, Rao AN, Ramesha MS, Raman A (2017). A global analysis of alternative tillage and crop establishment practices for economically and environmentally efficient rice production. Sci. Rep..

[31] Kumar V (2018). Can productivity and profitability be enhanced in intensively managed cereal systems while reducing environmental footprint of production? Assessing sustainable intensification options in breadbasket of India. Agric. Ecosyst. Environ..

[32] Jat HS (2018). Assessing soil properties and nutrient availability under conservation agriculture practices in a reclaimed sodic soil in cereal**-**based systems of North**-**West India. Arch. Agron. Soil Sci..

[33] Choudhary KM (2018). Evaluating alternatives to rice**-**wheat system in western Indo-Gangetic Plains: crop yields, water productivity and economic profitability. Field Crops Res..

[34] Nath CP, Das TK, Bhattacharyya R, Pathak H, Paul S, Chakraborty D, Hazra KK (2019). Nitrogen effects on productivity and soil properties in conventional and zero tilled wheat with different residue management. Proc. Nat Acad. Sci. India Sec. B: Bio. Sci..

[35] Mondal S, Poonia S, Mishra JS, Bhatt BP, Rao KK, Saurabh K, Kumar R (2019). Effect of short-term (5 years) conservation agriculture on soil physical properties and organic carbon under cereal based cropping system in Indo-Gangetic Plain of Bihar. Euro. J. Soil Sci..

[36] Jiang XJ, Liu S, Zhang H (2017). Effects of different management practices on vertical soil water flow patterns in the Loess Plateau. Soil Till. Res..

[37] Singh AK, Das B, Mali SS, Bhavana P, Shinde R, Bhatt BP (2019). Intensification of rice-fallow cropping systems in the Eastern Plateau region of India: diversifying cropping systems and climate risk mitigation. Climate Dev..

[38] Yadav GS (2017). Energy budgeting for designing sustainable and environmentally clean/ safer cropping systems for rainfed rice fallow lands in India. J. Clean. Prod..

[39] Bohra JS, Kumar R (2015). Effect of crop establishment methods on productivity, profitability and energetics of rice (*Oryza sativa*)-wheat (*Triticum aestivum*) system. Indian J. Agric. Sci..

[40] Gao B, Ju X, Meng Q, Cui Z, Christie P, Chen X, Zhang F (2015). The impact of alternative cropping systems on global warming potential, grain yield and ground water use. Agric. Ecosyst. Environ..

[41] Pishgar-Komleh SH, Ghahderijani M, Sefeedpari P (2012). Energy consumption and CO_2_ emissions analysis of potato production based on different farm size levels in Iran. J. Clean. Prod..

[42] Simmonds MB (2014). Seasonal methane and nitrous oxide emission of several rice cultivars in direct-seeded systems. J. Environ. Qual..

[43] Adviento-Borbe MAA, Haddix ML, Binder DL, Walters DT, Dobermann A (2007). Soil greenhouse gas fluxes and global warming potential in four high yielding maize systems. Global Change Biol..

