# Supplementary material for: Crop rotation and tillage management options for sustainable intensification of rice-fallow agro-ecosystem in eastern India
Source: Sci Rep. 2020 Jul 7;10:11146. doi: 10.1038/s41598-020-67973-9 (PMC7341809; doi:10.1038/s41598-020-67973-9)
Supplement: Supplementary file 1 — Supplementary file1 (DOCX 27 kb) [file 41598_2020_67973_MOESM1_ESM.docx]

**Crop rotation and tillage management options for sustainable intensification of rice-fallow agro-ecosystem in eastern India**

Rakesh Kumar^a*^, Janki Sharan Mishra^a*^, Karnena Koteswara Rao^a^, Surajit Mondal^a^, Kali Krishna Hazra^b^, Jaipal Singh Choudhary^c*^, Hansraj Hans^a^, Bhagwati Prasad Bhatt^a^

^a^ ICAR**-**Research Complex for Eastern Region, Patna**-**800 014, Bihar, India

^b^ ICAR**-**Indian Institute of Pulses Research, Kanpur**-**208 024, Uttar Pradesh, India

^c^ICAR**-**RCER Research Centre, Plandu, Ranchi**-**834 010, Jharkhand, India

*Corresponding authors’ email: [rakeshbhu08@gmail.com](mailto:rakeshbhu08@gmail.com) (Rakesh Kumar); [choudhary.jaipal@gmail.com](mailto:choudhary.jaipal@gmail.com) (Jaipal Singh Choudhary);

[jsmishra31@gmail.com](mailto:jsmishra31@gmail.com) (Janki Sharan Mishra)

**Suppl. Figure** **1.** Monthly average minimum and maximum temperatures (^o^C), rainfall (mm), and pan-evaporation (mm) during 2016-2017 and 2017-2018.

**Suppl. Table 1.** Initial soil physico-chemical properties of the experimental field.

| Parameter | Value | Method | Reference |
| --- | --- | --- | --- |
| Sand (%) | 10.7 | Hydrometer method | Bouyoucos^45^ |
| Silt (%) | 53.3 |  |  |
| Clay (%) | 36.0 |  |  |
| Texture | Silty clay loam |  |  |
| Bulk density (g cm^-3^) | 1.63 | Core Sampler | Piper^46^ |
| EC (dsm^-1^ at 25^o^C) | 0.01 | Conductivity bridge | Jackson^47^ |
| pH | 7.58 | Glass electrode pH meter | Jackson^47^ |
| Organic carbon (g kg^-1^) | 5.60 | Walkley and Black method | Walkley and Black^48^ |
| Available N (kg ha^-1^) | 183 | Alkaline permanganate method | Subbiah and Asija^49^ |
| Available P (kg ha^-1^) | 51 | Olsen’s method | Olsen *et al.*^50^ |
| Available K (kg ha^-1^) | 250 | Flame photometer | Hanway and Heidel^51^ |
| Available Fe (ppm) | 71.3 | EDTA* | Lindsay and Norvell^52^ |
| Available Zn (ppm) | 0.74 | EDTA | Lindsay and Norvell^52^ |
| Available Mn (ppm) | 12.39 | EDTA | Lindsay and Norvell^52^ |
| Available Cu (ppm) | 3.45 | EDTA | Lindsay and Norvell^52^ |

*EDTA Ethylene diamine tetra acetic acid

Bouyoucos, G.J. Hydrometer method for making particle size analysis of soil. *Agron. J.* **54**, 464**-**465 (1962).

Piper, C.S. Soil and Plant Analysis. Hans Publisher, Bombay (1966).

Jackson, M.L. Soil Chemical Analysis, Prentice Hall of India Ltd, New Delhi, 183**-**204 (1973).

Walkley, A. & Black, C.A. An examination of Degtjareff method for determining soil organic matter and proposed modification of chromic acid titration method. *Soil Sci.* **37**, 29**-**38 (1934).

Subbiah, B.V. & Asija, G.L. A rapid procedure for estimation of available nitrogen in soils. *Current Sci.* **28**, 259**-**260 (1973).

Olsen, S.R., Cole, C.V., Walanable, F.S. & Dean, L.A. Estimation of available phosphorus in soil by extraction with sodium bicarbonate. [Fine Methods of Soil Analysis : Agronomy No. 9, Black, C.A. (Ed), AmeSoc Agron, Inc., Madison, Wisconsin] USDA, Circular **939**, 19**-**23 (1954).

Hanway, J.J. & Heidel, H. Soil analysis methods as used in Iowa state college soil testing laboratory. *Iowa Agric.* **57**, (1952).

Lindsay, W.L. & Norvell, W.A. Development of a DTPA soil test for zinc, iron, manganese, and copper. *Soil Sci. Soc. Am. J.* **42**, 421**-**428 (1978).

**Suppl. Table 2.** Minimum support price (MSP) during the experimentation.

| Crops | Minimum support price (INR t^-1^) | |
| --- | --- | --- |
|  | 2016-17 | 2017-18 |
| Rice | 14700 | 15500 |
| Chickpea | 40000 | 44000 |
| Lentil | 39500 | 42500 |
| Safflower | 37000 | 41000 |
| Linseed | 50000 | 55000 |
| Mustard | 37000 | 40000 |

**Suppl. Table 3.** Energy equivalents of inputs and outputs in agricultural production.

| Particular | Unit | Equivalent energy (MJ) | Reference |
| --- | --- | --- | --- |
| **Inputs** | | | |
| Human Labour |  |  |  |
| Adult men | hour | 1.96 | Shahin *et al.* (2008) |
| Women | hour | 1.57 | Shahin *et al.* (2008) |
| Diesel | litre | 56.31 | Shahin *et al.* (2008) |
| Farm machinery | kwh | 62.7 | Shahin *et al.* (2008) |
| Chemical fertilizers | | | |
| N | kg | 60.6 | Shahin *et al.* (2008) |
| P_2_O_5_ | kg | 11.1 | Shahin *et al.* (2008) |
| K_2_O | kg | 6.70 | Shahin *et al.* (2008) |
| Farm yard manures (FYM) | kg (dry mass) | 0.30 | Shahin *et al.* (2008) |
| Electricity* | kWh | 11.93 | Mobtaker et al. (2010) |
| Water for irrigation | m^3^ | 1.02 | Shahin *et al.* (2008) |
| Plant protection (Superior) |  |  |  |
| Granulated chemical | kg | 120 | Gündoğmuş (2006) |
| Liquid chemical | litre | 120 | Gündoğmuş (2006) |
| **Outputs/grains/seeds** | | | |
| Rice, lentil, chickpea | kg | 14.7 | Shahin *et al.* (2008) |
| Mustard/linseed/safflower | kg | 25.0 | Shahin *et al.* (2008) |
| Straw/stover of cereal/pulses/oilseed | kg | 12.5 | Shahin *et al.* (2008) |

* 1-HP electric motor consumes 0.746 kW, so a 15-HP motor for pumping water consumes 15 × 0.746 = 11.2 kW of energy in 1 h.

Gündoğmuş, E. Energy use on organic farming: a comparative analysis on organic versus conventional apricot production on small holdings in Turkey. *Energ Convers Manage*. **47**, 3351-3359 (2006).

Mobtaker, H.G., Keyhani, A., Mohammadi, A., Rafiee, S.H. & Akram, A. Sensitivity analysis of energy inputs for barley production in Hamedan Province of Iran. *Agric. Ecosyst. Environ.*, **137**, 367-372 (2010.).

Shahin, S., Jafai, A., Mobli, H., Rafiee, S. & Karini M. Effect of farm size on energy ration on wheat production: a case study from Ardabil province of Iran. *Am.-Eurasian J Agric Environ Sci.* **3**, 604–608 (2008).
